# A Fluorescence Enhancement Sensor Based on Silver Nanoclusters Protected by Rich-G-DNA for ATP Detection

**DOI:** 10.3390/molecules29184490

**Published:** 2024-09-21

**Authors:** Yuxia Li, Jingxuan Ren, Zeting Meng, Baozhu Zhang

**Affiliations:** Department of Chemistry and Chemical Engineering, Jinzhong University, Yuci, Jinzhong 030619, China; yxli_2004@163.com (Y.L.); rjx2474488237@163.com (J.R.); vm2017111125@jzxy.edu.cn (Z.M.)

**Keywords:** fluorescence sensors, silver nanoclusters, ATP, aptamer

## Abstract

In this study, a turn-on fluorescence sensor for the detection of adenosine 5′-triphosphate (ATP) was developed and tested using ATP-DNA2-Ag NCs. The results showed that the fluorescence of ATP-DNA2-Ag NCs was significantly enhanced with the addition of ATP. The fluorescence enhancement was a result of the specific binding activity of the ATP aptamer and ATP, which caused G-rich sequences to approach the dark DNA-Ag NCs, owing to the alteration in ATP aptamer conformation. The proposed sensor demonstrated a good linear range of 18–42 mM and a limit of detection (LOD) of 2.8 μM. The sensor’s features include sensitivity, selectivity, and simple operation. In addition, the proposed sensor successfully measured ATP in 100-fold diluted fetal bovine serum.

## 1. Introduction

Metal nanoclusters (NCs) (e.g., Cu NCs, Ag NCs, and Au NCs) consist of several to hundreds of atoms with sizes of less than 2 nm. They have attracted attention for their application as fluorescence nanoprobes due to their excellent water dispersibility, size adjustability, and unique optical properties, such as discrete energy levels, a Fermi wavelength of electrons, high quantum yields, and good photostability [1,2,3,4]. Among NCs, silver nanoclusters protected by oligonucleotides (DNA-Ag NCs) display greater advantages compared with quantum dots or organic fluorophores, such as low toxicity, biocompatibility, an adjustable light emission wavelength, a large Stokes shift, and easy synthesis [5,6,7]. Recently, various analytes have been detected using DNA-Ag NCs, such as metal ions [8,9,10,11], small molecules [12,13], enzymes [14,15,16], DNA [17,18,19], proteins [20,21,22], etc.

ATP is an energy molecule in living organisms [23] that plays an important role in regulating intracellular metabolism and biochemical pathways in diverse organs and tissues [24,25]. Moreover, ATP supplies energy for different types of biochemical reactions in most living beings [26]. Additionally, ATP is regarded as a potential biomarker for ischemia [27], hypoglycemia [28], Parkinson’s disease [29], Alzheimer’s disease, hypoxia [30], and some malignant tumors [31]. Therefore, various types of ATP detection methods have been investigated, such as colorimetric methods [32], electrochemical analysis [33], fluorescence analysis [34], and liquid chromatography [35,36]. However, these methods are time-consuming and require laborious modification of electrodes and fluorescent probes to prepare complex samples. In recent years, various probes for ATP detection have been developed by our team. These probes are based on the principle that a significant increase in fluorescence occurs when the complementary linkers bring dark DNA-Ag NCs closer together [37]. The detection strategies employing DNA-Ag NCs [38,39,40] and DNA-Cu/Ag NCs [41] possess some advantages, including simple operation, high sensitivity, and selectivity. However, some of the parameters, including LODs, are still unsatisfactory. Hence, it is very urgent to develop detection methods with superior performance.

**Scheme 1:**
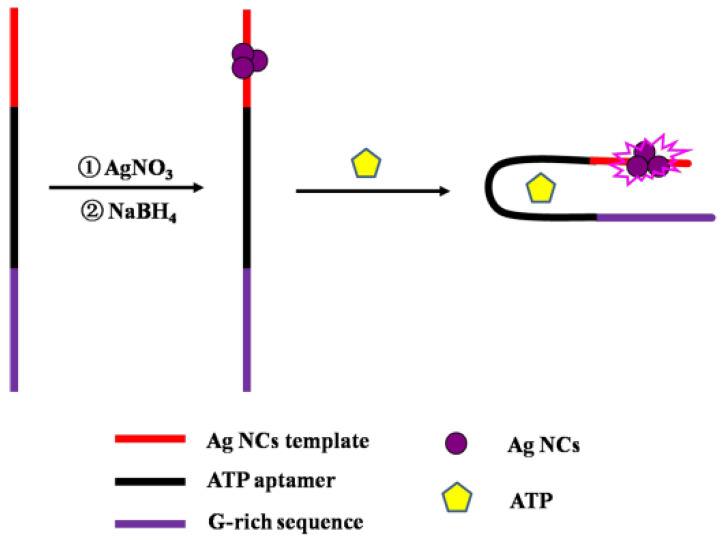
Schematic illustration of the sensor for ATP detection utilizing DNA-Ag NCs.

**Figure 1:**
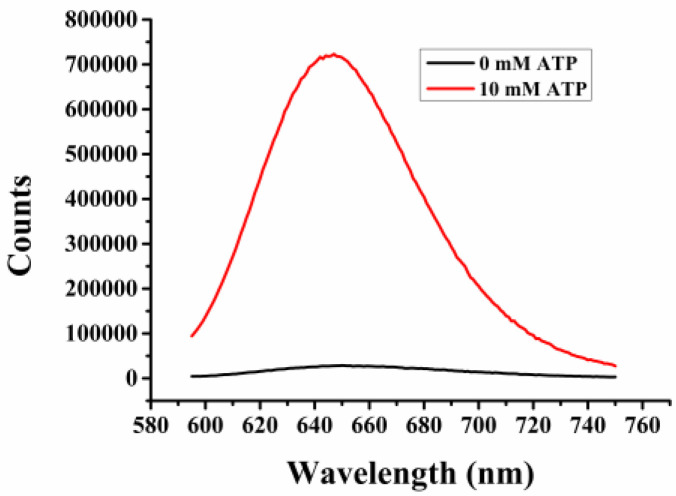
Feasibility for the detection of ATP.

Inspired by the fact that non-emissive DNA-Ag NCs become bright emitters as soon as a guanine-rich (G-rich) DNA sequence is close [37], a strategy was constructed employing DNA-Ag NCs with an ATP aptamer and G-rich sequences. Scheme 1 shows the sensor mechanism for ATP detection. The DNA template comprised the ATP aptamer in the middle, Ag NC-nucleation sequences located at 5′ ends, and G-rich sequences at 3′ ends. The darkish DNA-Ag NCs at 5′ ends become significantly brighter due to a conformational alteration in the ATP aptamer in the presence of ATP, bringing the G-rich sequences at 3′ ends closer together. Furthermore, a feasibility assay was carried out. As shown in Figure 1, the fluorescence of DNA-Ag NCs displayed obvious enhancement in the presence of 10 mM of ATP. Therefore, the proposed sensor not only features turn-on activation, sensitivity, and convenience, but it is also green.

## 2. Results and Discussion

### 2.1. Assay of Excitation and Emission Spectra, Stability, and Quantum Yield of DNA-Ag NCs

**Figure 2:**
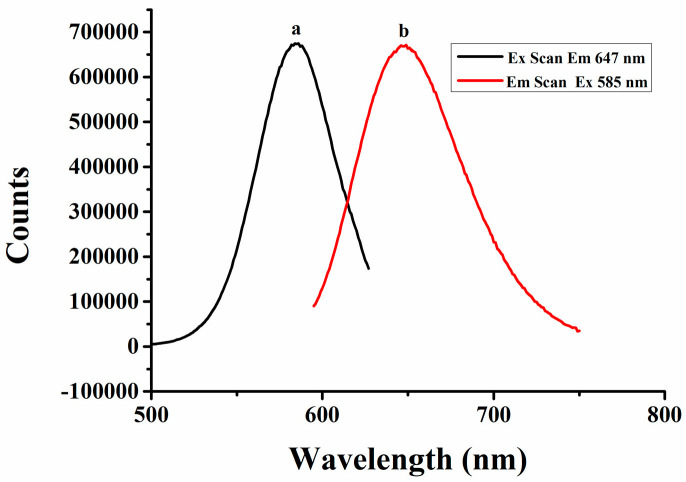
The excitation (a) and emission (b) spectra of ATP-DNA2-Ag NCs.

The secondary structures and base sequences of DNA templates play important roles in determining the optical properties of DNA-Ag NCs [42,43]. Due to their different structures and compositions, DNA-Ag NCs are determined by different secondary structures and base sequences of DNA templates, leading to various properties of DNA-Ag NCs, such as excitation and emission wavelengths and quantum yield. Therefore, four DNA templates named ATP-DNA, ATP-DNA1, ATP-DNA2, and ATP-DNA3 were designed. The DNA sequences are listed in Table 1. ATP-DNA is the basic template, which is composed of an ATP aptamer located in the middle part (bold), the C-rich sequences at its 5′ ends (italic), and TTTTT linkers (underlined) that connect the aptamer and the C-rich sequences. ATP-DNA1, ATP-DNA2, and ATP-DNA3 are derived from ATP-DNA, with G-rich sequences at their 3′ ends (red), and ATP-DNA3 has more TGGGG than ATP-DNA2 at its 3′ ends. Similarly, ATP-DNA2 also has more TGGGG than ATP-DNA1. Curves a and b in Figure 2 are the excitation and emission spectra of ATP-DNA2-Ag NCs; 585 nm and 647 nm are the excitation and emission wavelengths, respectively. Similarly, Supplementary Information Figure S1 presents the excitation and emission spectra of ATP-DNA-Ag NCs, ATP-DNA1-Ag NCs, and ATP-DNA3-Ag NCs as follows: excitation wavelengths of 550 nm, 580 nm, and 585 nm and emission wavelengths of 611 nm, 644 nm, and 649 nm. Additionally, the UV–vis absorption spectra of ATP-DNA2-Ag NCs were obtained with the addition of 0, 6, 12, 18, and 24 mM of ATP. Two obvious peaks can be observed in each curve of Supplementary Information Figure S2. The peaks at 430 nm and 585 nm were determined to belong to the characteristic plasmon absorption bands of Ag nanoparticles and ATP-DNA2-Ag NCs [39], respectively. Therefore, ATP-DNA2-Ag NCs contain many Ag NCs and a small number of Ag nanoparticles (NPs). The enhancement of the absorption intensity demonstrates that the amount of ATP-DNA2-Ag NCs increases with increasing ATP concentration.

Because the detection performance is influenced by the stability of the sensor, the emission intensities of the four DNA-Ag NCs against incubation time were determined. As shown in Supplementary Information Figure S3, the emission intensity (Supplementary Information Figure S3A) of ATP-DNA2-Ag NCs slowly declined and remained stable after 300 min. The emission intensity (Supplementary Information Figure S3B) of ATP-DNA-Ag NCs gradually enhanced for the first 90 min; then, it remained stable for about 120 min, slowly declining thereafter. Similar change trends were found for ATP-DNA1 and ATP-DNA3-Ag NCs, with their emission intensities (Supplementary Information Figure S3C,D) gradually increasing at first and then sharply declining. The emission intensity (Supplementary Information Figure S3C) of ATP-DNA1-Ag NCs declined slower than ATP-DNA3-Ag NCs. Although the emission intensity of ATP-DNA-Ag NCs can remain stable for 2 h, the results of ATP detection in ATP-DNA-Ag NCs are not sufficient. Therefore, improving the stability of ATP-DNA-Ag NCs is still an important research topic in the future. Finally, taking into account various factors comprehensively, the ATP-DNA2-Ag NCs were selected as the most suitable candidates for the subsequent experiments in this study.

To evaluate the ability of the tested NCs to emit fluorescence, the quantum yield (QY) values of the ATP-DNA-Ag NCs, ATP-DNA1-Ag NCs, ATP-DNA2-Ag NCs, and ATP-DNA3-Ag NCs sensors were detected. As shown in Supplementary Information Figure S4, QY results were 76.91%, 28.39%, 86.33%, and 9.39%, respectively. Although the C-rich-sequences of the NCs were the same, the microenvironments were different due to different G-rich-sequences at 3′ ends. Moreover, the microenvironments of the ATP-DNA2-Ag NCs may be superior to the other NCs. Therefore, the QY of the ATP-DNA2-Ag NCs was determined to be the highest.

### 2.2. TEM Images and XPS Measurements of ATP-DNA2-Ag NCs

**Figure 3:**
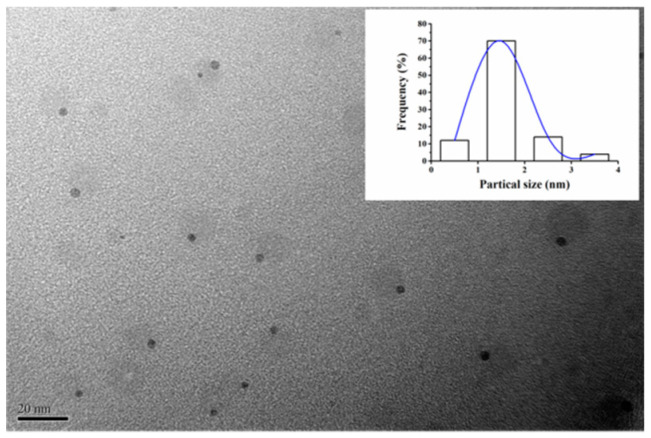
TEM image of ATP-DNA2-Ag NCs (inset: size distribution histogram).

TEM images of ATP-DNA2-Ag NCs were obtained to investigate the NCs’ morphologies and sizes. In Figure 3, the uniform distribution of ATP-DNA2-Ag NCs can be observed, and the average diameter is less than 2 nm, which is in accordance with the size (not more than 2 nm) of metal NCs [44]. These data were obtained from a histogram employing the Lorentzian function.

**Figure 4:**
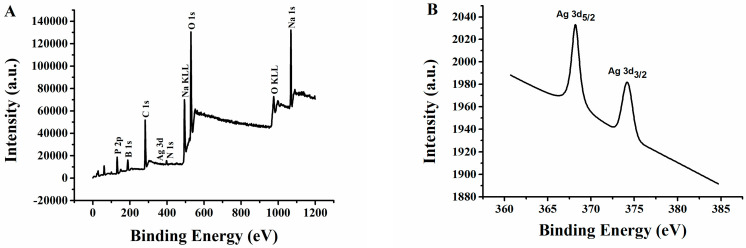
(**A**) XPS spectrum of ATP-DNA2-Ag NCs. (**B**) Ag 3d region of the XPS spectrum of ATP-DNA2-Ag NCs.

To identify the Ag elements and valence states that exist in ATP-DNA2-Ag NCs, X-ray photoelectron spectroscopy (XPS) was conducted. As shown in Figure 4A, Ag, B, C, N, O, Na, and P were found to exist in ATP-DNA2-Ag NCs. In addition, the expanded spectrum of Ag 3d was found to exist, as indicated by the two peaks shown in Figure 4B, whose binding energies were 368.2 eV for Ag 3d_5/2_ and 374.2 eV for Ag3d_3/2_, which are attributed to Ag (1) and Ag (0) in ATP-DNA2-Ag NCs [45,46], respectively. Therefore, Ag (1) and Ag (0) exist in ATP-DNA2-Ag NCs.

### 2.3. Optimization of the Assay Conditions

#### 2.3.1. Effect of DNA Templates on the Optical Properties of DNA-Ag NCs

DNA-Ag NCs scaffolded using different DNA templates have different properties [42,43]. Moreover, the properties of DNA-Ag NCs directly affect ATP detection. Hence, the fluorescence variation in ATP-DNA1-Ag NCs, ATP-DNA2-Ag NCs, and ATP-DNA3-Ag NCs without and with 10 mM of ATP was measured. As shown in Supplementary Information Figure S5, the fluorescence intensities of the NCs remarkably increased with 10 mM of ATP. However, the relative fluorescence intensity (*F*/*F*_0_, *F*_0_, and *F* are the maximum emission intensities of DNA-Ag NCs before and after the addition of 10 mM of ATP) of ATP-DNA2-Ag NCs increased most obviously by more than 25 times. Apparently, ATP-DNA2-Ag NCs perform better than the other two DNA-Ag NCs. This may be the reason that the amount of G bases of ATP-DNA2 at its 3′ ends is the most suitable because the amount is between that of ATP-DNA1 and ATP-DNA3. Therefore, ATP-DNA2-Ag NCs show the best performance when the G-rich sequences at 3′ ends approach the dark Ag NCs at 5′ ends, with the addition of ATP. Whereas ATP-DNA1-Ag NCs contain a lesser amount of G bases, ATP-DNA3-Ag NCs contain a little more G bases. Therefore, the ATP-DNA2-Ag NCs were selected as the most suitable candidate for detecting ATP.

#### 2.3.2. Determination of Reaction Time

The reaction time between ATP-DNA2-Ag NCs and 10 mM of ATP was determined since it has a certain effect on ATP detection. As illustrated in Supplementary Information Figure S6, the fluorescence intensity of ATP-DNA2-Ag NCs gradually increased at first, reaching a maximum after 5 min, and then remained unchanged for 1 h. Therefore, 5 min was selected as the most suitable reaction time for subsequent experiments.

#### 2.3.3. Effect of pH on the Optical Properties of ATP-DNA2-Ag NCs

The relative fluorescence intensity (*F*/*F*_0_) of ATP-DNA2-Ag NCs upon increasing pH values from 5.0 to 9.0 was measured due to the effects of pH on the optical properties of ATP-DNA2-Ag NCs. Supplementary Information Figure S7 shows that the *F*/*F*_0_ of ATP-DNA2-Ag NCs reached a maximum value at pH 7.0 and then declined from pH 7.0 to 9.0. Thus, PBS with pH 7.0 was used for all of the experiments.

### 2.4. Measurement of ATP in PBS

**Figure 5:**
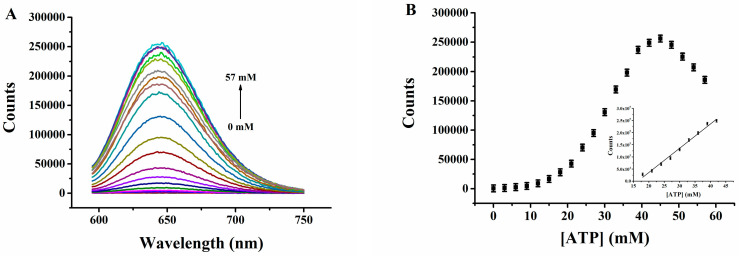
(**A**) Fluorescence emission spectra of ATP-DNA2-Ag NCs in the presence of ATP (0–57 mM). (**B**) ATP concentration-dependent changes of fluorescence intensity at 647 nm. Inset: Linear range between *F*_647 nm_ and ATP concentration (18–42 mM). Error bars represent the standard deviation of three repetitive experiments.

Sensitivity is an important parameter for an effective sensor. Therefore, ATP (0–57 mM) was detected using the proposed sensor under optimal conditions. Figure 5A,B show that the emission intensity of ATP-DNA2-Ag NCs gradually enhanced with increasing ATP concentration. A good linear relationship within 18–42 mM is shown in Figure 4B (*R*^2^ = 0.9889, the linear regression equation is *F* = −163,821.5 + 9979.2*C_ATP_*). The limit of detection (LOD) was as low as 2.8 μM, which was calculated based on 3*σ*_0_/*k* (the value of *σ*_0_ was obtained from the standard deviation of the background, and *k* represents the slope of the calibration line). Supplementary Information Table S2 shows that the LOD of the proposed sensor is far lower than that of the fluorescence sensors previously reported by the authors of [38,41,45,47]. Therefore, the proposed sensor has high sensitivity. In addition, compared to fluorescence detection methods based on distyrylbenzene (1) with two arms of dipicolylaminomethyl groups at the central benzene ring and its zinc complex (1-Zn) [34], rhodamine derivatives [48], and labeled aptamer with magnetic nanoparticles [49], these methods have been applied to analyze ATP with good sensitivity and selectivity. However, the first two types of probes require complex organic synthesis, while the latter requires labeling 6-carboxyfluorescein onto the aptamer. These operations are not only complex but also may affect the detection effect. Moreover, the biocompatibility of organic probes is also poor. Therefore, the proposed method performs superior to other methods.

The fluorescence lifetime of ATP-DNA2-Ag NCs was investigated at an emissive wavelength of 647 nm upon the addition of 0, 5, 10, 15, and 20 mM of ATP (Supplementary Information Figure S8). As shown in Supplementary Information Table S2, the fluorescence transient of ATP-DNA2-Ag NCs present haploid exponential time constants, and the average lifetime is 3.48 ns. Supplementary Information Table S2 displays that the lifetime of ATP-DNA2-Ag NCs slightly fluctuated at 3.50 ns with increasing ATP concentration. Thus, the interaction between ATP-DNA2-Ag NCs and ATP is the result of a static process.

### 2.5. Interference Study

**Figure 6:**
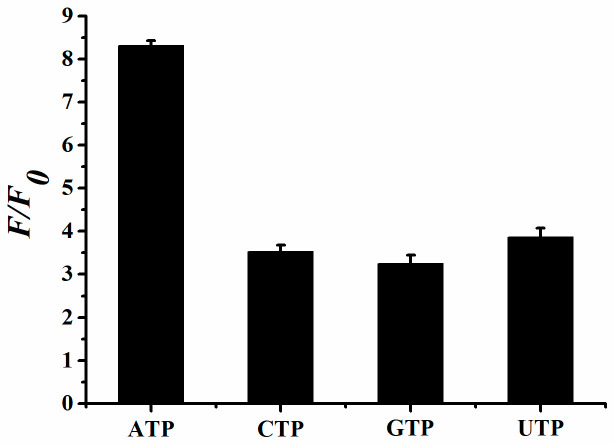
Selectivity of the proposed sensor. The concentrations of ATP, CTP, GTP, and UTP were 10 mM. Error bars represent the standard deviation of three repeated experiments.

The selectivity of the proposed sensor toward ATP was evaluated by utilizing other nucleoside triphosphates, including CTP, UTP, and GTP. As shown in Figure 6, the *F*/*F*_0_ of ATP-DNA2-Ag NCs was 8.3, 3.5, 3.2, and 3.6 when 10 mM amounts of ATP, CTP, GTP, and UTP were added, respectively. It was apparent that the proposed sensor caused dramatic enhancement in *F*/*F*_0_ in the presence of ATP, whereas other interferents resulted in only a slight enhancement in *F*/*F*_0_. The high specificity of this detecting system might be attributed to the fact that ATP has a high binding affinity to its adapter. Hence, the result of the interference study demonstrates that the proposed sensor is highly selective toward ATP.

### 2.6. Investigation of ATP in Diluted Fetal Bovine Serum

The practicability of the proposed sensor is also an important factor. Hence, ATP-DNA2-Ag NCs were employed to detect ATP spiked in 100-fold diluted fetal bovine serum. Table 2 shows that the recoveries varied from 99.5 to 101.7% with relative standard deviations (RSDs) of 0.95–1.18%. The results indicate that it possesses high precision, which is comparable with the previous values [50,51]. Therefore, the results demonstrate that the proposed sensor is expected to be successfully applied to ATP detection in actual samples.

## 3. Experimental Section

### 3.1. Reagents and Instruments

Oligonucleotides, adenosine 5′-triphosphate (ATP), cytidine 5′-triphosphate (CTP), guanosine 5′-triphosphate (GTP), and uridine 5′-triphosphate (UTP) were purchased from Sangon Biotechnology Inc. (Shanghai, China). The sequences and names of the oligonucleotides are listed in Table 1. Silver nitrate (AgNO_3_, 99.8%) and sodium borohydride (NaBH_4_, 98%) were obtained from Aladdin Bio-chem Technology Co., Ltd. (Shanghai, China). Fetal bovine serum was acquired from Yuanye Biotechnology Co., Ltd. (Shanghai, China). Phosphate buffered solution (PBS, pH 7.0, 20 mM) was used in all experiments. All chemical reagents were of analytical grade. Milli-Q water (18.2 MΩ cm) was used to prepare all solutions.

The absorption spectra were detected using a Cary 50 Bio spectrophotometer (Varian Inc., Palo Alto, CA, USA) at ambient temperature. Fluorescence spectra were measured using an Edinburgh FS5 fluorescence spectrophotometer (Edinburgh Instruments, Livingston, UK) at room temperature, and the slit widths for excitation and emission were 2.0 nm and 4.0 nm, respectively. Time-resolved fluorescence measurements were carried out using an FS5 fluorescent lifetime spectrometer (Edinburgh Instruments, Livingston, UK) operating in the time-correlated single photon counting (TCSPC) mode using a semiconductor laser (405 nm) as the excitation source. Commercial software by Edinburgh Instruments was utilized for data analyses. When $\sum_{i=1}^{n}A_{i}$ = 1, the average excited state lifetime is expressed by the equation τ_avg_ = $\sum_{i=1}^{n}A_{i}\tau_{i}$. The reported spectrum of each sample represents the average of three scans. A JEOL JEM-2100 (Tokyo, Japan) transmission electron microscope with 200-kV acceleration voltage was utilized to obtain the morphologies and average size of the Ag NCs. X-ray photoelectron spectroscopy (XPS) (ESCAL-ab 220i-XL, VG Scientific, London, UK) was performed using a monochromic Al Ka source at 1486.6 eV.

### 3.2. Preparation of DNA-Ag NCs

**Figure 7:**
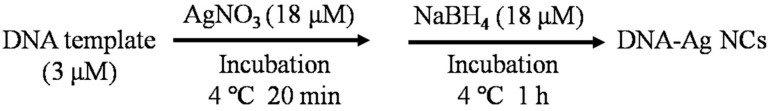
The synthetic route for DNA-Ag NCs.

The DNA-Ag NCs were synthesized using a method previously reported in the literature [40]. Briefly, 3.0 μM of DNA and 18 μM of Ag NO_3_ were uniformly mixed with 20 mM of PBS (pH 7.0). Then, the above mixture solution was kept in the dark at 4 °C for 20 min. After 20 min, a freshly prepared solution of NaBH_4_ (18 μM) was added to the abovementioned mixture solution and promptly stirred for 1 min. Then, the samples were incubated for 1 h under dark conditions at 4 °C. Finally, the prepared DNA-Ag NCs were obtained and stored for future use. In addition, Figure 7 was the synthetic route for DNA-Ag NCs.

### 3.3. Sensing Experiments in PBS

An amount of 0–57 mM of ATP was added to the DNA-Ag NCs solution samples. The fluorescence spectrum of each sample was detected after incubation for 5 min at ambient temperature. To determine the specificity of the assay system toward ATP, the other nucleoside triphosphates, such as GTP, CTP, and UTP, were tested in experiments employing the same method used to evaluate ATP.

### 3.4. Application of the Sensor

Fetal bovine serum was selected as the real sample for the detection of ATP. Briefly, ATP was separately spiked into 100-fold diluted fetal bovine serum at final concentrations ranging from 15 to 24 mM and detected by employing the same method previously used for ATP detection.

### 3.5. Detection of Absolute Photoluminescence Quantum Yield

The absolute photoluminescence quantum yield (APLQY) of the proposed sensing system was detected based on diffuse reflectance and absorbance spectra employing the SC-30 Integrating Sphere Module from an FS5 Sample Module. Additionally, *η* = *N^em^/N^abs^* is its mathematical formula (*η* represents the absolute quantum yield, which is obtained by the number of photons emitted divided by the number of photons absorbed). Furthermore, *η* was calculated via “direct excitation” measurements, where the emission and scatter of a sample are recorded when the radiation is directly excited from the excitation monochromator alone.

## 4. Conclusions

In conclusion, in this study, we developed an assay system based on the fluorescence enhancement of ATP-DNA2-Ag NCs caused by the approach of G-rich sequences, which was utilized as a sensor for the sensitive and selective detection of ATP. The LOD of the proposed sensor was 2.8 μM, with a range of 18–42 mM. Furthermore, the proposed sensor demonstrated satisfactory results in the detection of ATP in diluted fetal bovine serum. The assay system possesses advantages, including simplicity, specificity, sensitivity, low cost, and hypotoxicity. Moreover, the system does not require chemical modification and thus provides a promising method for ATP detection in biological fields.

## Figures and Tables

**Table 1:** Names and sequences of the oligonucleotides.

Oligonucleotids	Sequences (5′-3′)
ATP-DNA	*CCCTTAATCCCC* TTTTT **AACCTGGGGGAGTATTGCGGAGGAAGG** TTTTT
ATP-DNA1	*CCCTTAATCCCC* TTTTT **AACCTGGGGGAGTATTGCGGAGGAAGG** TTTTT GGGTGGGG
ATP-DNA2	*CCCTTAATCCCC* TTTTT **AACCTGGGGGAGTATTGCGGAGGAAGG** TTTTT GGGTGGGGTGGGG
ATP-DNA3	*CCCTTAATCCCC* TTTTT **AACCTGGGGGAGTATTGCGGAGGAAGG** TTTTT GGGTGGGGTGGGGTG**GGG**

**Table 2:** Determination of ATP in diluted fetal bovine serum solution (*n* = 3).

Samples	Spiked (mM)	Measured (mM) Means ^a^ ± SD ^b^	Average Recovery (%)	RSD (%)
1	15	15.2 ± 0.18	101.3	1.18
2	18	18.3 ± 0.19	101.7	1.04
3	21	20.9 ± 0.22	99.5	1.05
4	24	24.3 ± 0.23	101.3	0.95

^a^ The mean of three determinations. ^b^ Standard deviation.

## Data Availability

Data are contained within the article and Supplementary Materials.

## Supplementary Materials

The following supporting information can be downloaded at https://www.mdpi.com/article/10.3390/molecules29184490/s1. Table S1: Comparison of different strategies for the detection of ATP; Table S2: The lifetimes of ATP-DNA2-Ag NCs in the absence and presence of ATP; Figure S1: The excitation (curve a) and emission (curve b) spectra (A, B, and C) of ATP-DNA-Ag NCs, ATP-DNA1-Ag NCs, and ATP-DNA3-Ag NCs; Figure S2: UV-vis absorption spectra of ATP-DNA2-Ag NCs under different concentrations of ATP; Figure S3: The change in fluorescence intensity of ATP-DNA2, ATP-DNA, ATP-DNA1, and ATP-DNA3-Ag NCs (A, B, C, and D) against increases in incubation time. Error bars represent the standard deviation of three independent measurements. *c*(DNA) = 3.0 μM; Figure S4: The absolute photoluminescence quantum yield (APLQY) of ATP-DNA, ATP-DNA1, ATP-DNA2, and ATP-DNA3-Ag NCs (A, B, C, and D); Figure S5: Relative fluorescence intensity (*F/F*_0_) of different DNA-Ag NCs. *F*_0_ and *F* are the maximum emission intensities of the DNA-Ag NCs before and after the addition of 10 mM of ATP, respectively. The error bars represent the standard deviation of three independent measurements; Figure S6: Fluorescence intensity of ATP-DNA2-Ag NCs as a function of incubation time of ATP-DNA2-Ag NCs and ATP. The error bars represent the standard deviation of three independent measurements; Figure S7: Relative fluorescence intensity (*F/F*_0_) of ATP-DNA2-Ag NCs at different pH values. *F*_0_ and *F* are the maximum emission intensities of ATP-DNA2-Ag NCs before and after adding 10 mM ATP, respectively. The error bars represent the standard deviation of three independent measurements; Figure S8: The fluorescence lifetimes of ATP-DNA2-Ag NCs (excitation at 405 nm and emission at 625 nm) incubating without and with the different concentration of ATP.
