# Supplementary material for: A Fluorescence Enhancement Sensor Based on Silver Nanoclusters Protected by Rich-G-DNA for ATP Detection
Source: Molecules. 2024 Sep 21;29(18):4490. doi: 10.3390/molecules29184490 (PMC11433816; doi:10.3390/molecules29184490)
Supplement: Supplementary file 1 [file molecules-29-04490-s001.zip › molecules-3197425-supplementary.pdf]

Electronic Supplementary Information

A Fluorescence Enhancement Sensor Based on Silver Nanoclusters Protected by Rich-G-DNA for ATP Detection

Yuxia Li, Jingxuan Ren, Zeting Meng and Baozhu Zhang *

Department of Chemistry and Chemical Engineering, Jinzhong University, Yuci, Jinzhong 030619, China; yxli_2004@163.com (Y.L.); rjx2474488237@163.com (J.R.); vm2017111125@jzxy.edu.cn (Z.M.)

* Correspondence: zhangbaozhu518@126.com

Table of contents

Table S1 Comparison of different strategies for the detection of ATP. S3

Table S2 The lifetimes of ATP-DNA2-Ag NCs in the absence and presence of ATP. S3

Figure S1. The excitation (curve a) and emission (curve b) spectra (A, B, C) of ATP-DNA-Ag NCs, ATP-DNA1-Ag NCs and ATP-DNA3-Ag NCs. S3

Figure S2. UV-vis absorption spectra of ATP-DNA2-Ag NCs under different concentrations of ATP. S4

Figure S3. The change in fluorescence intensity of ATP-DNA2, ATP-DNA, ATP-DNA1, and ATP-DNA3-Ag NCs (A, B, C, and D) *against* increases in incubation time. Error bars represent the standard deviation of three independent measurements. *c*(DNA) = 3.0 μM. S4

Figure S4. The absolute photoluminescence quantum yield (APLQY) of ATP-DNA, ATP-DNA1, ATP-DNA2 and ATP-DNA3-Ag NCs (A, B, C, and D). S5

Figure S5. Relative fluorescence intensity (F/F_0) of different DNA-Ag NCs. F_0 and F are the maximum emission intensities of the DNA-Ag NCs before and after the addition of 10 mM of ATP, respectively. The error bars represent the standard deviation of three independent measurements. S5

Figure S6. Fluorescence intensity of ATP-DNA2-Ag NCs as a function of incubation time of ATP-DNA2-Ag NCs and ATP. The error bars represent the standard deviation of three independent measurements. S5

Figure S7. Relative fluorescence intensity (F/F_0) of ATP-DNA2-Ag NCs at different pH values. F_0 and F are the maximum emission intensity of ATP-DNA2-Ag NCs before and after adding 10 mM ATP, respectively. The error bars represent the standard deviation of three independent measurements. S6

Figure S8. The fluorescence lifetimes of ATP-DNA2-Ag NCs (excitation at 405 nm and emission at 625 nm) incubating without and with the different concentration of ATP. S6

Table S1 Comparison of different strategies for the detection of ATP.

Detection methods	LOD	Linear range	References
DNA-Ag NC fluorescence light-up system	0.44 mM	0-4 mM	38
Fluorescence DNA-Cu/Ag NCs	7.0 µM	2-18 mM	41
Fluorescence-based core–shell Ag@SiO_2 nanoflares	8 µM	0-500 µM	45
Light-up DNA-scaffolded silver nanoclusters	0.81 mM	1-6 mM	47
Fluorescence DNA-Ag NCs	2.8 µM	18-42 mM	this work

Table S2 The lifetimes of ATP-DNA2-Ag NCs in the absence and presence of ATP.

Samples	[ATP] (mM)	τ (ns)	χ^2
ATP-DNA2-Ag NCs + ATP	0	3.50	1.268
	5	3.44	1.200
	10	3.52	1.287
	15	3.51	1.249
	20	3.45	1.209

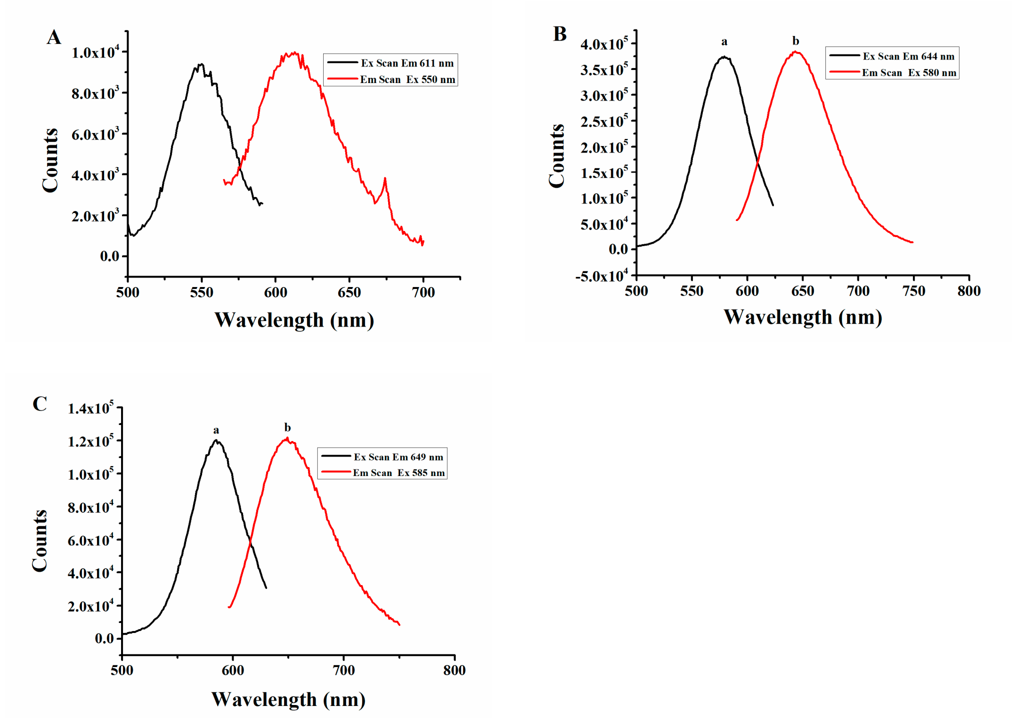

Figure S1. The excitation (curve a) and emission (curve b) spectra (A, B, and C) of ATP-DNA-Ag NCs, ATP-DNA1-Ag NCs and ATP-DNA3-Ag NCs.

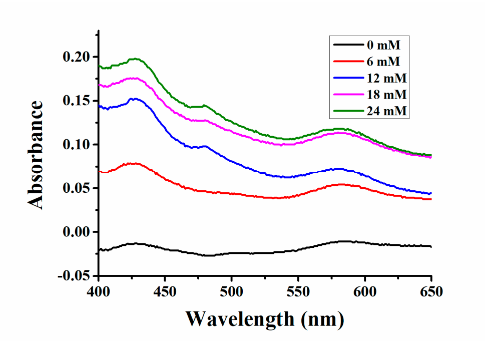

Figure S2. UV-vis absorption spectra of ATP-DNA2-Ag NCs under different concentrations of ATP.

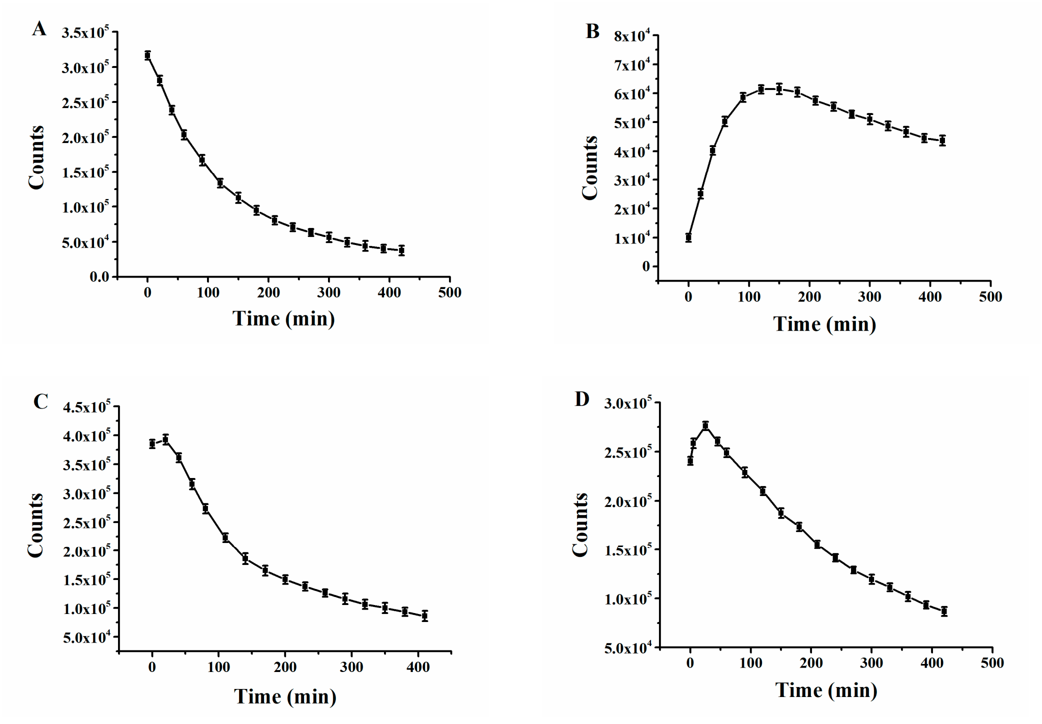

Figure S3. The change of fluorescence intensity of ATP-DNA2, ATP-DNA, ATP-DNA1, and ATP-DNA3-Ag NCs (A, B, C, and D) *against* increases in incubation time. Error bars represent the standard deviation of three independent measurements. c(DNA) = 3.0 μM.

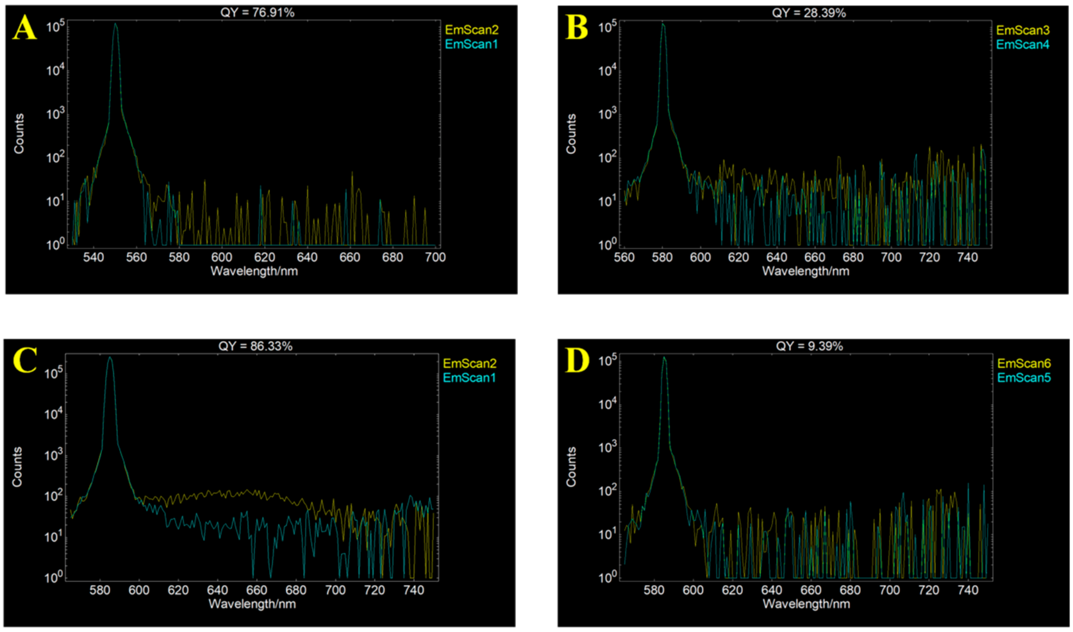

Figure S4. The absolute photoluminescence quantum yield (APLQY) of ATP-DNA, ATP-DNA1, ATP-DNA2 and ATP-DNA3-Ag NCs (A, B, C, and D).

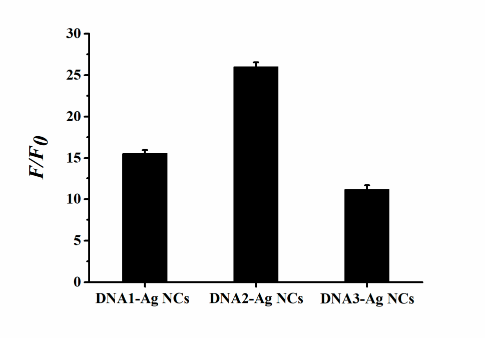

Figure S5. Relative fluorescence intensity (F/F_0) of different DNA-Ag NCs. F_0 and F are the maximum emission intensities of the DNA-Ag NCs before and after the addition of 10 mM of ATP, respectively. The error bars represent the standard deviation of three independent measurements.

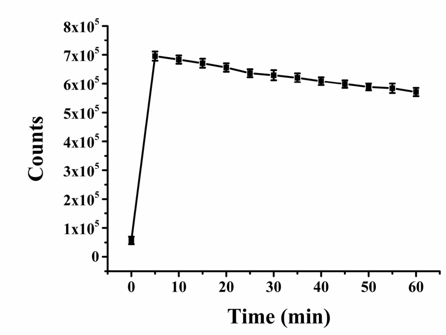

Figure S6. Fluorescence intensity of ATP-DNA2-Ag NCs as a function of incubation time of ATP-DNA2-Ag NCs and ATP. The error bars represent the standard deviation of three independent measurements.

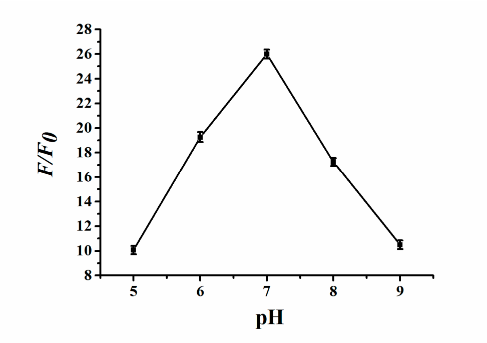

Figure S7. Relative fluorescence intensity (F/F_0) of ATP-DNA2-Ag NCs at different pH values. F_0 and F are the maximum emission intensities of ATP-DNA2-Ag NCs before and after adding 10 of mM ATP, respectively. The error bars represent the standard deviation of three independent measurements.

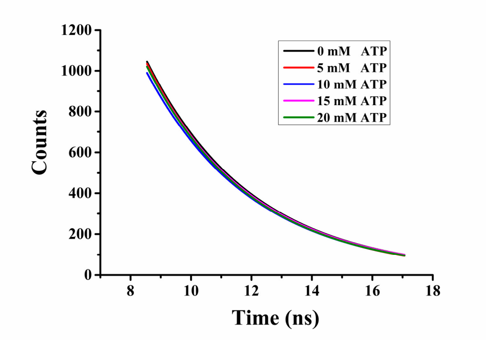

Figure S8. The fluorescence lifetimes of ATP-DNA2-Ag NCs (excitation at 405 nm and emission at 647 nm) incubating without and with the different concentrations of ATP.
